# Complexity of skeletal muscle degeneration: multi-systems pathophysiology and organ crosstalk in dystrophinopathy

**DOI:** 10.1007/s00424-021-02623-1

**Published:** 2021-09-22

**Authors:** Kay Ohlendieck, Dieter Swandulla

**Affiliations:** 1 Department of Biology, Maynooth University, National University of Ireland, Co. Kildare, Maynooth, W23F2H6 Ireland; 2 Kathleen Lonsdale Institute for Human Health Research, Maynooth University, Co. Kildare, Maynooth, W23F2H6 Ireland; 3 Institute of Physiology, University of Bonn, 53115 Bonn, Germany

**Keywords:** Dystrophin, Duchenne muscular dystrophy, Fibrosis, Inflammation, Muscle degeneration, Organ crosstalk

## Abstract

Duchenne muscular dystrophy is a highly progressive muscle wasting disorder due to primary abnormalities in one of the largest genes in the human genome, the *DMD* gene, which encodes various tissue-specific isoforms of the protein dystrophin. Although dystrophinopathies are classified as primary neuromuscular disorders, the body-wide abnormalities that are associated with this disorder and the occurrence of organ crosstalk suggest that a multi-systems pathophysiological view should be taken for a better overall understanding of the complex aetiology of X-linked muscular dystrophy. This article reviews the molecular and cellular effects of deficiency in dystrophin isoforms in relation to voluntary striated muscles, the cardio-respiratory system, the kidney, the liver, the gastrointestinal tract, the nervous system and the immune system. Based on the establishment of comprehensive biomarker signatures of X-linked muscular dystrophy using large-scale screening of both patient specimens and genetic animal models, this article also discusses the potential usefulness of novel disease markers for more inclusive approaches to differential diagnosis, prognosis and therapy monitoring that also take into account multi-systems aspects of dystrophinopathy. Current therapeutic approaches to combat muscular dystrophy are summarised.

## Introduction

Contractile tissues in general, and skeletal muscle fibres in particular, occupy a special position in the physiological systems of the human body, making up approximately 40% of body weight. Voluntary contractile fibres and their associated cell types display a remarkable array of special features on various levels of biological organisation ranging from genotype to phenotype [226]. Although enormous progress has been made in the elucidation of the underlying mechanisms of myogenesis [44, 307] and muscle plasticity [262, 263], various fundamental questions of skeletal muscle physiology remain to be fully resolved [273]. Many of the functional and structural specialisations of the muscular system play body-wide roles in health and disease, affecting especially locomotion, posture, heat homeostasis and metabolic networks and their integration [50]. Given this context, the complexity and multifunctionality of the constituents of the skeletal muscle proteome is reflected by the diversity of muscular disorders [81]. In addition to neurological, metabolic and autoimmune diseases that indirectly affect the motor system, intrinsic disorders of skeletal muscles manifest as inflammatory myopathies, myotonias, congenital myopathies, pharmacogenetic myopathies and muscular dystrophies [71, 340]. The current list of neuromuscular disorders includes over 1,000 individual pathologies with over 600 identified genes that are associated with monogenic neuromuscular disorders [21], whereby many of these muscular disorders are already diagnosed at young age [70]. The most frequently inherited primary muscle disease of early childhood is Duchenne muscular dystrophy (DMD) [197], a highly progressive disorder of voluntary contractile fibres [88, 98, 215] that is associated with a high level of caregiver burden and illness costs [289].

On the level of the skeletal musculature, genetic defects in the *DMD* gene cause the almost complete loss of the membrane cytoskeletal protein dystrophin, which causes progressive symmetrical muscle wasting, in combination with sterile inflammation, fat substitution and reactive myofibrosis. A variety of recent reviews provide excellent details on the discovery of dystrophin [130], the genetic basis of dystrophinopathy [84, 117, 228], the complexity of pathophysiological mechanisms that underlie the muscle-related pathogenesis [6, 141, 300, 319, 340], diagnosis and clinical management of Duchenne patients [25–27, 215] and novel therapeutic strategies to treat progressive muscle degeneration and associated complications in X-linked muscular dystrophy [101, 298, 299]. This review builds on this accumulated knowledge on dystrophinopathy with a focus on the concept that Duchenne muscular dystrophy displays multi-systemic abnormalities. This biomedical idea is especially based on recent findings generated by large-scale analyses of both genetic disease models and patient specimens. Therefore, this article attempts to provide an inclusive overview of the molecular and cellular aspects that lead to highly complex skeletal muscle degeneration in association with a multi-systems pathogenesis and organ crosstalk in X-linked inherited muscular dystrophy [111, 324]. Following an introduction into the organisation of the *DMD* gene, its tissue-specific expression pattern and the tight interactions within dystrophin complexes (which is crucial for our understanding of the role of the various dystrophin isoforms in multiple tissue and organ systems), the structure and function of the various protein products of the dystrophin gene and their pathophysiological role are examined.

Individual sections of this review outline crucial aspects of the differential effects of dystrophin deficiency on different skeletal muscles, late-onset cardio-respiratory complications and associated multi-system abnormalities including aberrant functioning of the nervous system, the liver, the kidney, the gastrointestinal tract and the immune system. Since systems biological approaches have been extensively applied to studying the molecular and cellular pathogenesis of muscular dystrophy and have resulted in the identification of complex biomarker signatures, the suitability and robustness of novel diagnostic and prognostic biomarker candidates of dystrophinopathy and organ crosstalk are presented. This includes the discussion of cell, tissue and organ disease markers in association with biofluid-related markers, as well as the evaluation of the potential for establishing therapeutic biomarkers of pharmacological interventions, cell-based approaches and gene therapies to counter-act dystrophin deficiency.

### The DMD gene, its expressed proteoforms and the genetic basis of dystrophinopathy

**Fig. 1:**
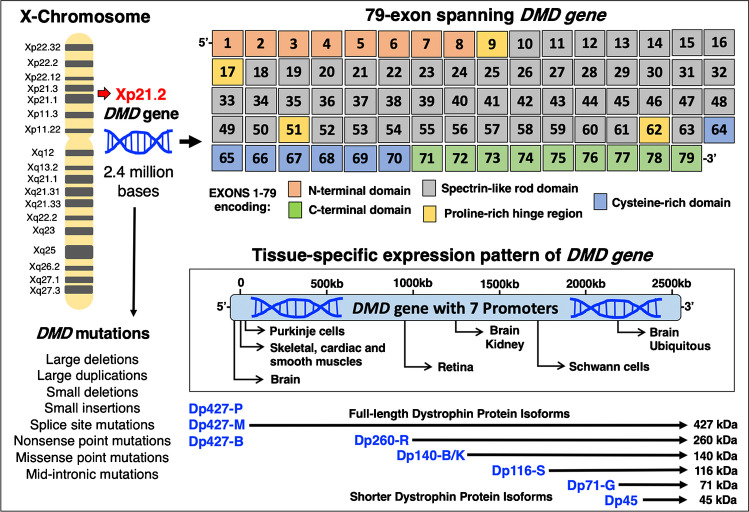
Overview of the *DMD* gene, its promoter structure and tissue-specific expression pattern of dystrophin isoforms. Abbreviations used: B, brain; B/K, brain/kidney; Dp, dystrophin protein; G, general; M, muscle; P, Purkinje cell; R, retina; S, Schwann cell

In skeletal muscle fibres, one of the largest genes in the human genome, the X-chromosomal 79-exon spanning *Dmd* gene [130, 164], exhibits the highest expression levels in form of the full-length Dp427-M isoform of the membrane cytoskeletal protein dystrophin [228]. The *DMD* gene is positioned on the short arm of the X-chromosome at the Xp21.2 band. As outlined in Fig. 1, the 79 exons encode an amino-terminal region with an actin-binding site, 4 proline-rich hinge regions, large central spectrin-like rod domains, a cysteine-rich domain and a carboxy-terminal domain with binding sites for various dystrophin-associated proteins [164–166]. The large number of distinct binding sites displayed by the protein product of the *DMD* gene provides the structural basis for a supramolecular dystrophin node at the sarcolemma [75], as outlined below. The tissue-specific expression of dystrophin isoforms is driven by seven different promoters. The protein products include three full-length dystrophins in brain, muscle and Purkinje cells, i.e. isoforms Dp427-B, Dp427-M and Dp427-P [228, 261] and shorter isoforms in the retina, brain/kidney, Schwann cells, the brain and a variety of other tissues, i.e. Dp260-R [349], Dp140-B/K [182, 183], Dp116-S [198, 206], Dp71-G [240] and Dp45 [278].

**Fig. 2:**
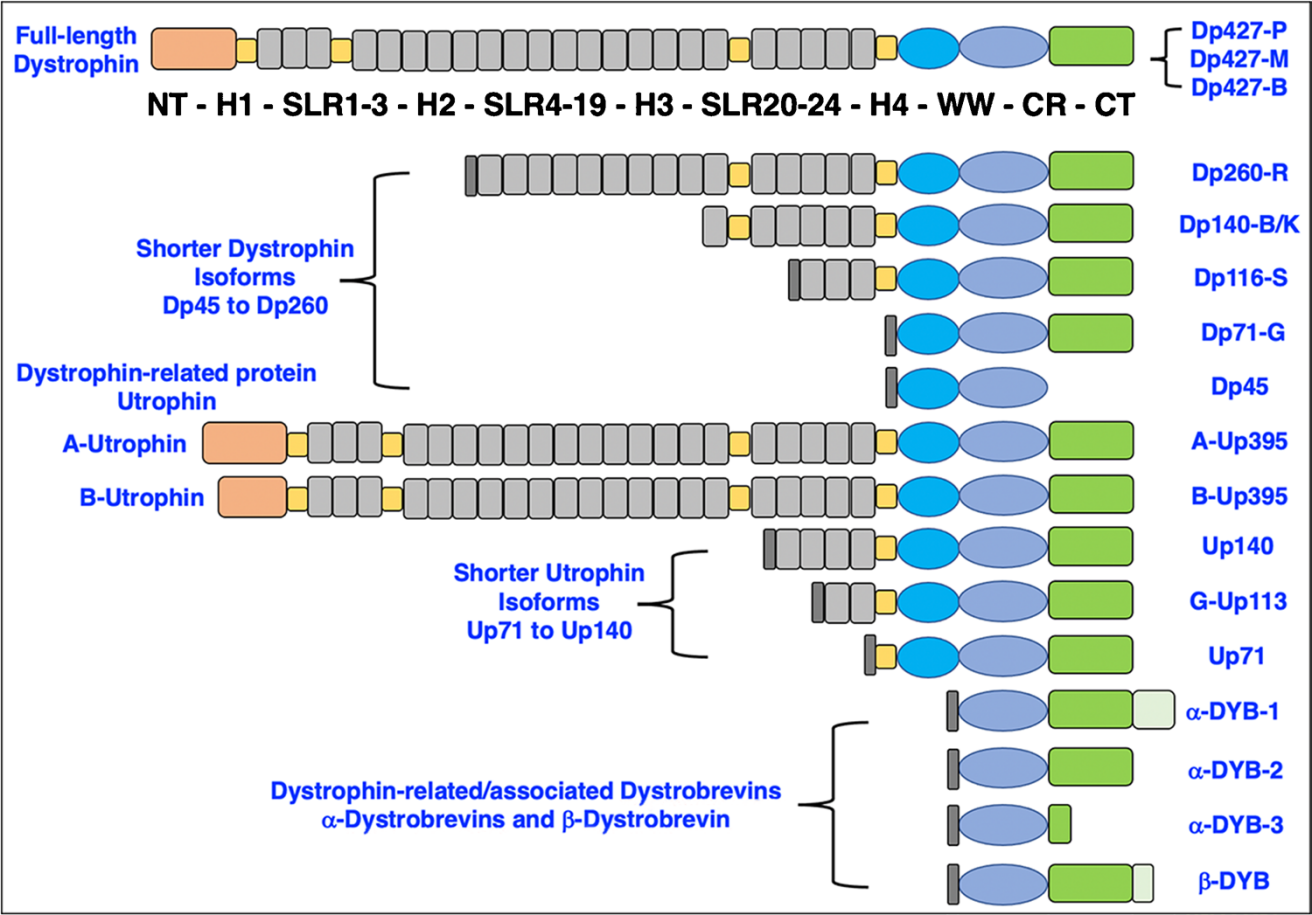
Domain structure of full-length dystrophin, shorter dystrophin isoforms and the main types of dystrophin-related proteins. Abbreviations used: B, brain; B/K, brain/kidney; CT, carboxy-terminus; CR, cysteine-rich domain; Dp, dystrophin protein; G, general; DYB, dystrobrevin; H, proline-rich hinge region; M, muscle; NT, amino-terminus; P, Purkinje cell; R, retina; S, Schwann cell; SLR, spectrin-like rod domain; WW, conserved region with signature tryptophan residues; Up, utrophin

The Dp427-M isoform belongs to the class of giant proteins [233] and was identified in all major types of contractile tissues, including skeletal muscle, cardiac muscle and smooth muscle [75, 134, 162]. An overview of the basic structure of the various isoforms of dystrophin, as compared to its autosomal homologue utrophin and the dystrobrevin family of proteins, is provided in Fig. 2. In contrast to the X-chromosomal *DMD* gene, the genes *UTRN*, *DTNA* and *DTNB* are autosomal and encode the dystrophin-related proteins utrophin, alpha-dystrobrevin and beta-dystrobrevin with the chromosomal locations 6q24, 18q12 and 2p24, respectively. Full-length utrophin of apparent 395 kDa is highly enriched in the neuromuscular junction [115, 251] and exists in several isoforms, including A-Up395, B-Up395, Up140, G-Up113 and Up71 [260, 342, 372]. Dystrobrevins were shown to exist as four distinct isoforms, i.e. alpha-DYB-1, alpha-DYB-2, alpha-DYB-3 and beta-DYB [29, 241, 271].

The complexity and enormous size of the *Dmd* gene with its 2.4-Mbp sequence requires considerable processing of the dystrophin 14-kb mRNA and a lengthy period for transcription [332, 346]. A large array of mutations and genetic rearrangements in the *Dmd* gene result in distinct effects on the various protein products, including abnormal size and/or amount of dystrophin isoforms in X-linked muscular dystrophy [84]. As listed in Fig. 1, primary genetic abnormalities in the *DMD* gene on the short arm of the X-chromosome include small and large deletions, small and large insertions, large duplications, missense point mutations, nonsense point mutations, splice site mutations and mid-intronic mutations [28, 99]. Diagnostic testing of these diverse primary abnormalities in the *DMD* gene can be routinely performed with a variety of genetic techniques [297], such as (i) diverse types of polymerase chain reaction assays [2] that mostly focus on the analysis of potential deletions [149], (ii) comparative genomic hybridisation array technology that can predict whether genetic changes may disrupt the reading frame [204], (iii) multiplex ligation-dependent probe amplification methods which are capable of swiftly assessing the copy number of exons and related genetic changes [292] and (iv) next-generation sequencing for the analysis of nonsense or missense types of point mutations, as well as small deletions [243, 255]. Genomic sequencing has also been applied to the detailed genetic characterisation of female carriers of the mutated *DMD* gene [368]. While the highly progressive Duchenne type of X-linked muscular dystrophy is characterised by genetic defects that result in the almost complete loss of the Dp427-M isoform of dystrophin in contractile tissues [30, 133], late-onset and less progressive Becker muscular dystrophy shows only reduced density and/or size of the dystrophin protein [170]. Hence, the difference between severe versus more benign forms of dystrophinopathy is based on the type of genetic alterations [297] and whether particular mutations cause the loss of dystrophin or the production of an abnormal but still semi-functional protein product [255].

### Association of dystrophin with other proteins

In the sub-sarcolemmal cytoskeleton of skeletal muscle fibres, the full-length isoform of dystrophin does not exist in isolation but forms a tightly associated membrane assembly [89, 90, 249, 252]. As recently reviewed, dystrophin can be considered an organising node of the muscle periphery [75]. A sub-complex consisting of dystrophin isoform Dp427-M and its associated integral glycoprotein beta-dystroglycan form the basis of a sarcolemma-spanning structure [147], which tightly interacts with the extracellular laminin-receptor alpha-dystroglycan, the integral glycoproteins alpha/beta/gamma/delta-sarcoglycan and the highly hydrophobic membrane component sarcospan, as well as the cytosolic proteins alpha/beta-dystrobrevin and alpha/beta-syntrophin [234]. This dystrophin core complex in turn provides a linkage to (i) the wider extracellular matrix (including laminin-211, fibronectin, biglycan and collagen isoforms COL-IV and COL-VI) [212, 230, 268], (ii) the intracellular cytoskeletal network (including cortical actin, cytokeratin, desmin, vimentin, tubulin, synemin and plectin) [23, 266, 274, 350], (iii) signalling proteins (such as the neuronal isoform nNOS of nitric oxide synthase, various kinases, the aquaporin water channel, the growth factor receptor-bound protein Grb2 and the insulin receptor, as well as crucial ion-regulatory proteins including Na^+^ channels, inward rectifier K^+^ channels, voltage-sensing L-type Ca^2+^ channels and transient receptor potential cation channels) [46, 103, 179, 290, 374] and (iv) the costamere structures (in conjunction with the mechano-sensing axis of integrin, vinculin and talin) of the fibre periphery [151].

**Fig. 3:**
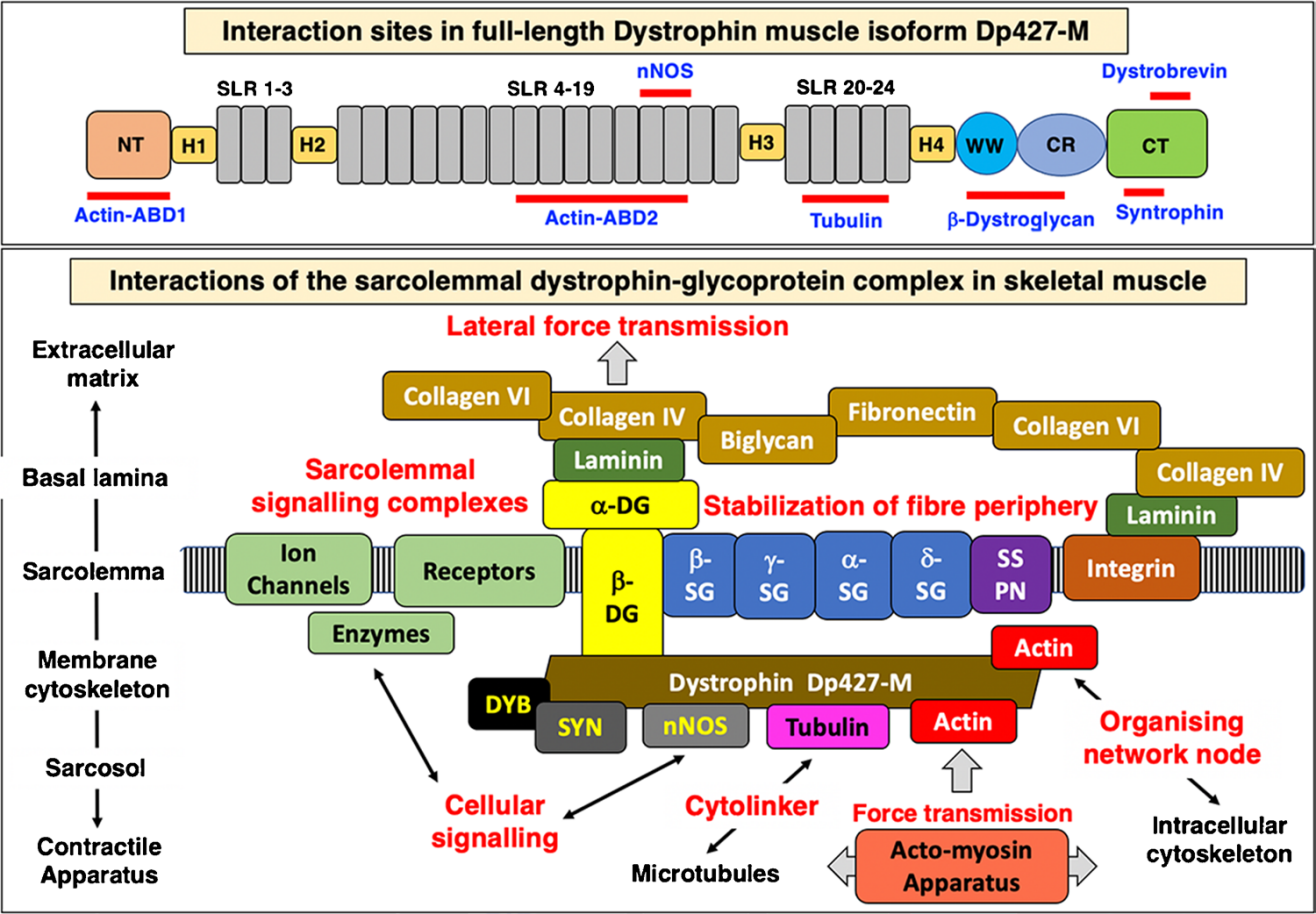
Interaction sites of dystrophin isoform Dp427-M and overview of the dystrophin-glycoprotein complex at the sarcolemma of skeletal muscle fibres. Abbreviations used: ABD, actin-binding domain; CT, carboxy-terminus; CR, cysteine-rich domain; DG, dystroglycan; DYB, dystrobrevin; Dp427-M, muscle-specific dystrophin isoform of 427 kDa; H, proline-rich hinge region; nNOS, neuronal isoform of nitric oxide synthase; NT, amino-terminus; SG, sarcoglycan; SLR, spectrin-like rod domain; SSPN, sarcospan; SYN, syntrophin; WW, conserved region with signature tryptophan residues

Thus, the dystrophin node functions as a central integrator of fibre stability, cytoskeletal organisation, cellular signalling and lateral force transmission [6, 51, 75, 179]. The discovery that dystrophin interacts directly with tubulin puts this membrane-associated protein into the class of cytolinkers [266]. Since besides dystrophin, the dystrophin-associated protein complex also plays a key role in the pathogenesis of dystrophinopathy [137], its core structure and diverse interconnectivity with the actin cytoskeleton, sarcolemma membrane, ion-regulatory components and the extracellular matrix is shown diagrammatically in Fig. 3. Besides in skeletal muscles, where the dystrophin complex was shown to form a monomeric structure of apparent 1.2 MDa size [287], this membrane-associated protein assembly also exists in the heart [154] and smooth muscle cells, such as the outer layers of the stomach wall [77]. However, the structure and subcellular localisation is slightly different as compared to skeletal muscle fibres. In cardiac muscle, the dystrophin complex was shown to be also present in the transverse tubular membrane system [154, 162] in contrast to the almost exclusive subcellular localisation in the sarcolemma membrane in skeletal muscles [252, 366, 385].

### Developmental stages of dystrophinopathy and skeletal muscle fibre degeneration

**Fig. 4:**
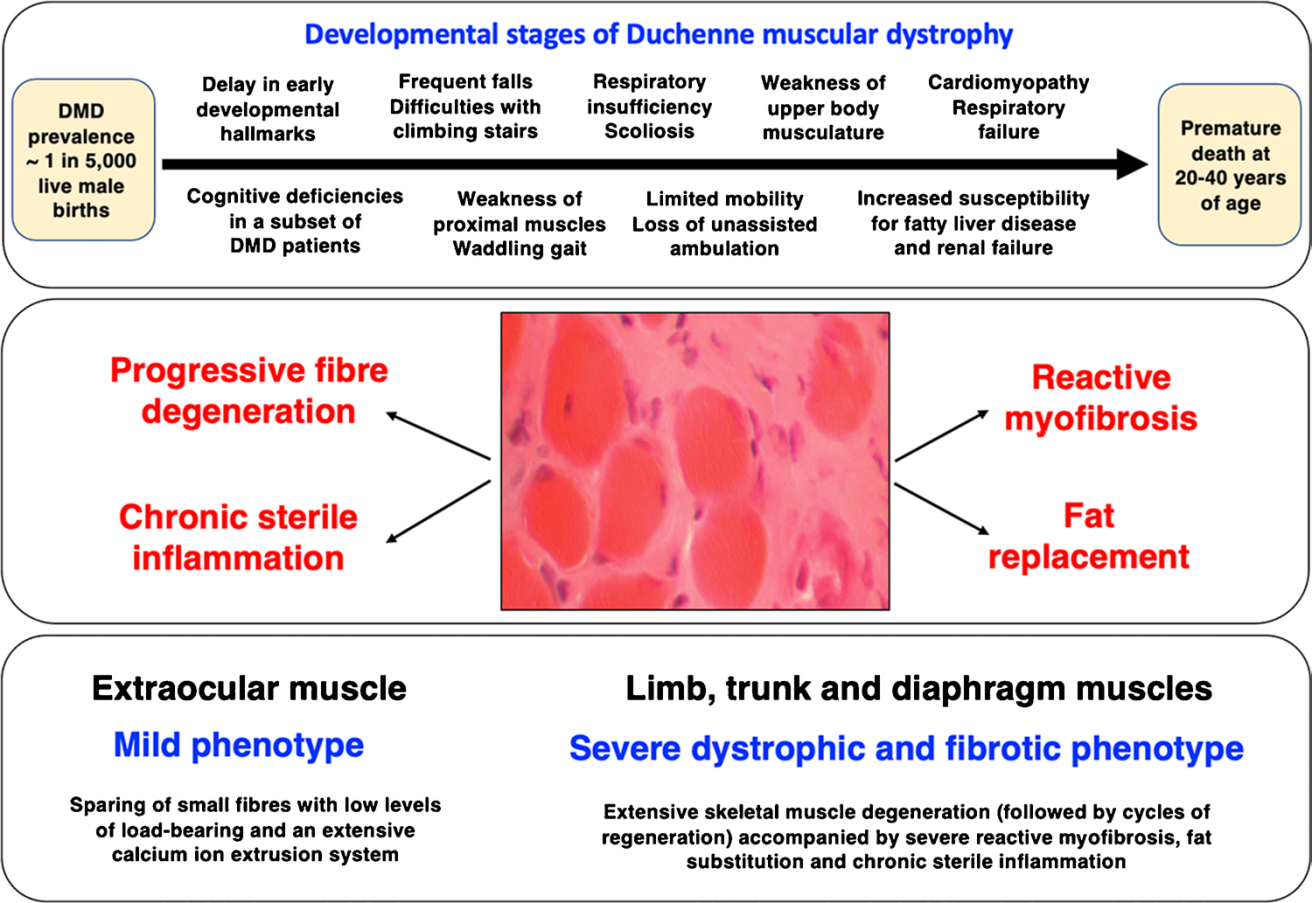
Summary of developmental stages of dystrophinopathy and muscle symptoms

Duchenne muscular dystrophy is the most common neuromuscular disorder of early childhood with a prevalence of approximately 1 in 5,000 live male births [52, 197] and is often initially detected due to developmental delay in conjunction with contractile weakness, slower walking and Gower’s sign, indicating weakness of proximal muscles [84, 98, 111]. Major clinical milestones of the developmental stages of dystrophinopathy are summarised in Fig. 4. The presence of Gower’s sign in Duchenne patients relates to proximal muscular weakness in hip and thigh muscles, which requires the help of both hands and arms for rightening the body to reach a standing position. Muscular dystrophy-associated temporal and spatial variations in gait were shown to include changes in cadence, anterior pelvic tilt and dorsiflexion during swing [68]. The proper assessment of pathological gait patterns and functional ambulation are crucial for prediction of disease progression, as well as monitoring of drug treatment and physiotherapeutic interventions [157]. Detailed studies of gait abnormalities have established a drastic decrease in walking speed, stride length, step length, maximal power generation at the hip, maximal knee extension torque, maximal dorsiflexion torque and maximal power generation at the ankle in Duchenne children [112, 113]. Common complications are hip, knee and ankle joint plantar flexion contractures [47]. Progressive muscle weakness also affects bone strength due to low bone mineral density in Duchenne patients [285]. This may cause an increased risk of bone fragility [25, 221], especially in association with prolonged glucocorticoid therapy [327, 365]. Key developmental stages of the disease include initially frequent falls, difficulties with climbing stairs, toe walking and a waddling gait, followed by progressive limitations in general mobility, respiratory insufficiency and scoliosis [105].

At a later stage, the loss of unassisted ambulation is followed by weakness of upper body musculature [25–27, 215]. Loss of ambulation as a clinical indicator of disease progression was shown to correlate with the type of genetic abnormality in the *DMD* gene. Of note, small mutations were associated with a younger age of loss of ambulation as compared to large deletions or duplications [19]. In the second decade of life, cardiomyopathy and respiratory failure requires ventilatory assistance and intervention with cardiac drug treatment [161, 216]. Duchenne patients also exhibit an increased susceptibility for fatty liver disease, gastrointestinal complications, renal failure, and bladder dysfunction [26, 200, 357]. Steady advances in cardiopulmonary care and pharmacological therapy have preserved quality of life and improved prognosis for survival of Duchenne patients in recent years. Premature death occurs usually at 20 to 40 years of age [27, 174]. However, cases of individual Duchenne patients living into their fifth and even sixth decade of life have been described [291, 360]. Especially the usage of oral corticosteroids has significantly prolonged ambulation in patients suffering from Duchenne muscular dystrophy, and therapy with angiotensin-converting enzyme inhibitors and beta-blockers was shown to delay the progression of cardiomyopathic complications [335]. The importance of ventilation is clearly supported by findings from a recent meta-analysis that has established differences in median life expectancy of 14–27 years versus 21–40 years in patients without versus with ventilatory support, respectively [174].

For the differential diagnostic evaluation of dystrophic patients, the above-described genetic tests are routinely utilised in conjunction with general physical examinations, motor and gait assessments, the evaluation of muscle biopsy specimens using histological and immunochemical tests [248] and serum assays focusing on the status of general muscle damage markers such as creatine kinase [237, 250]. In addition, magnetic resonance imaging presents a crucial non-invasive and multi-parametric assessment tool for the quantification of muscle pathology [4]. Muscle imaging correlates relatively well with histologic parameters [160] and can be employed for both diagnostic purposes and the extended monitoring of progressive alterations in the dystrophin-deficient skeletal musculature and clinical outcome measures [371]. Recently, the evaluation of increased collagen levels by multispectral optoacoustic tomography has been established and can be employed as an advanced imaging tool for the characterisation of reactive myofibrosis in association with dystrophinopathy [272]. The pathophysiological importance of fibrotic changes in dystrophinopathy is discussed in more detail in the below section.

**Fig. 5:**
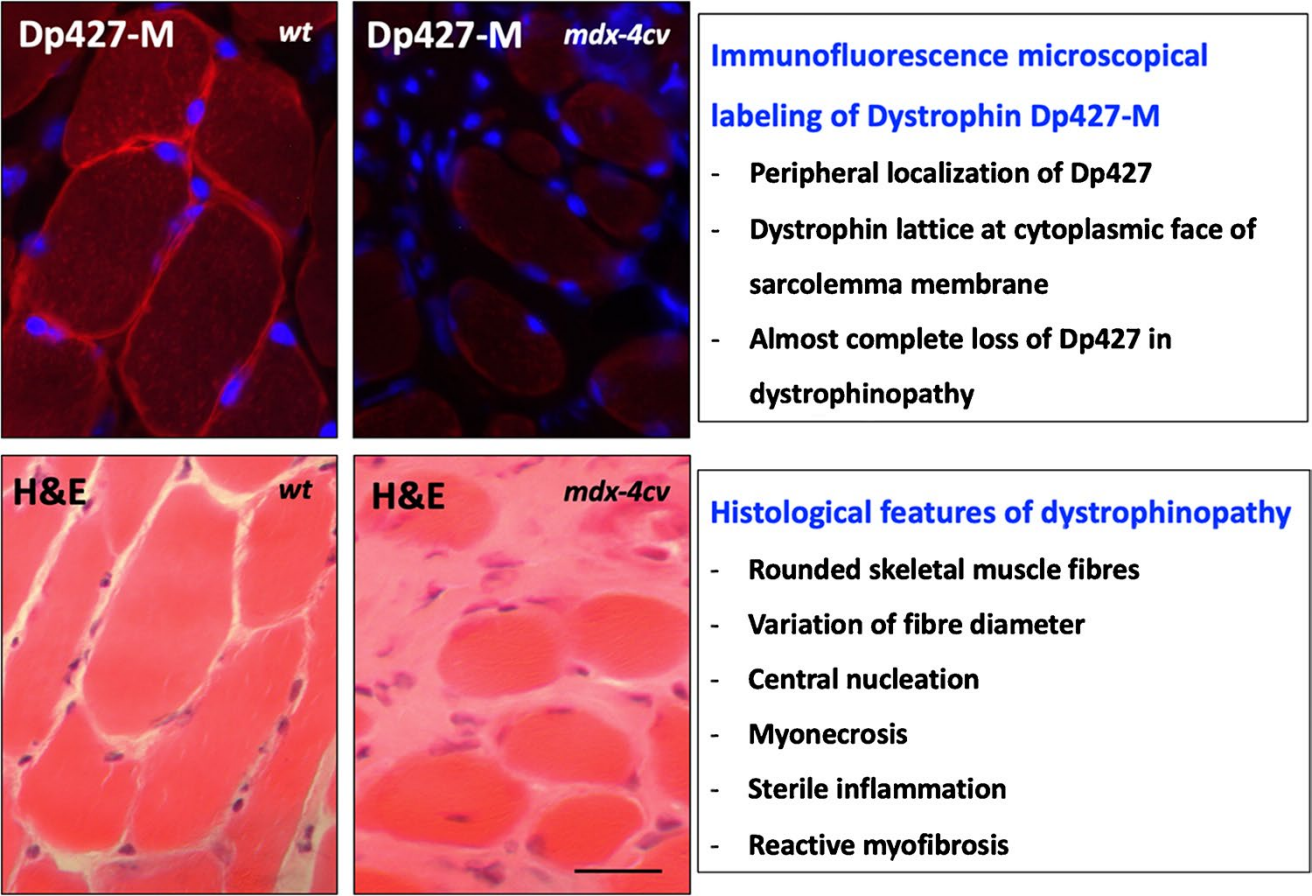
Absence of dystrophin isoform Dp427-M in X-linked muscular dystrophy and key histopathological features of dystrophinopathy. Shown is the immunofluorescence microscopical analysis (using monoclonal antibody NCL-DYS1 to dystrophin and Hoechst-33342 labelling of nuclei) and histological comparison (using haematoxylin and eosin staining; H&E) of skeletal muscle cryosections from wild type (*wt*) versus the *mdx-4cv* mouse model of Duchenne muscular dystrophy. Bar equals 40 μm

In progressive X-linked muscular dystrophy, the decline in skeletal muscle strength is reflected on the histological level by characteristic changes in fibre size, a more roundly appearance of myofibres and a high degree of central nucleation, as well as myonecrosis, clusters of inflammatory cells, hypercontractility, fibre branching, fatty deposition and myofibrosis. Figure 5 illustrates the subcellular localisation of dystrophin isoform Dp427-M in skeletal muscle using immunofluorescence microscopy. Full-length dystrophin exhibits a peripheral localisation [252, 385] whereby this membrane cytoskeletal protein is proposed to form a stabilising lattice at the cytoplasmic face of the sarcolemma membrane [366]. A striking feature of dystrophinopathy is the almost complete loss of dystrophin and a drastic reduction in all dystrophin-associated proteins in contractile fibres [253]. Dystrophin deficiency renders myofibers more susceptible to micro-rupturing of its plasmalemma and contraction-induced injury. Leaky surface membrane systems and impaired luminal calcium buffering [53] were shown to trigger impaired calcium handling [13, 72, 74], abnormal excitation–contraction coupling [39] and activation of calcium-dependent proteolytic degradation of muscle proteins [3, 144, 202].

Physiological dysregulation and enhanced proteolysis in dystrophic fibres are accompanied by a sustained cellular stress response and the upregulation of various chaperoning proteins to counteract proteotoxic insults to dystrophin-deficient fibres [35], especially small heat shock proteins such as alphaB-crystallin and the muscle-specific chaperone cvHSP [73]. The comprehensive mass spectrometric profiling of muscle biopsy samples from Duchenne patients has confirmed severe cytoskeletal and extracellular dysregulation in Dp427-deficient skeletal muscle [38].

**Fig. 6:**
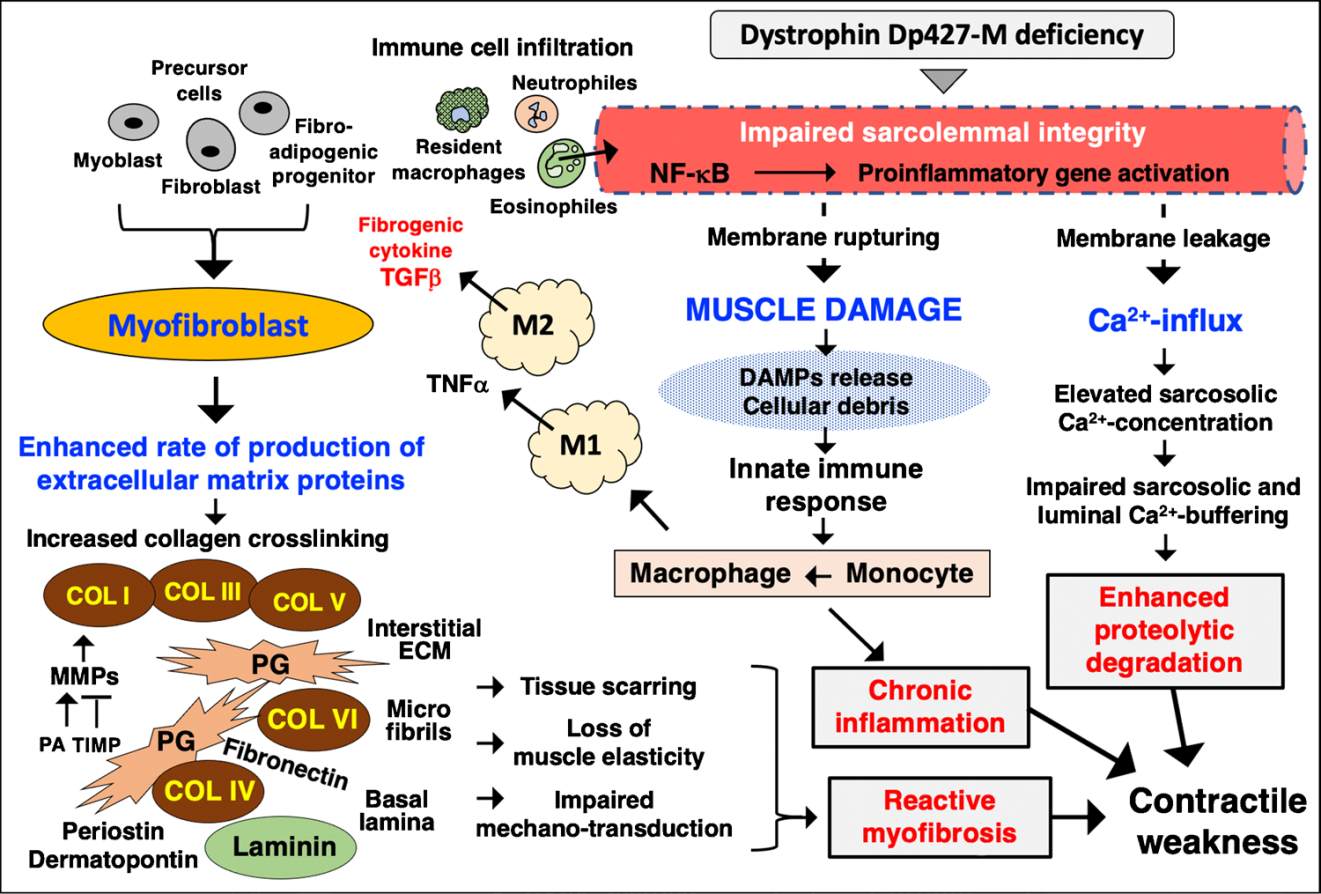
Pathophysiological role of chronic inflammation, reactive myofibrosis and abnormal calcium handling in the molecular and cellular pathogenesis of dystrophinopathy. Abbreviations used: COL, collagen; DAMPs, damage-associated molecular patterns; ECM, extracellular matrix; MMP, matrix metalloproteinase; PA, plasminogen activator; TIMP, tissue inhibitor of metalloproteinases; TGF, transforming growth factor; TNF, tumour necrosis factor; NF-κB, nuclear factor kappa B

The importance of abnormal calcium homeostasis in X-linked muscular dystrophy is summarised in Fig. 6, which also highlights the pathophysiological interconnectivity between the innate immune response to muscle damage and resulting activation of myofibroblasts and their role in reactive myofibrosis. In addition to dysregulated ion homeostasis, sterile inflammation and myofibrosis, the drastic reduction of the dystrophin-associated nNOS isoform of nitric oxide synthase in dystrophic fibres affects signalling between contractile cells and their microvasculature causing use-dependent muscle ischemia [339]. A recent study using Doppler sonography established that the severity of the dystrophic phenotype correlates with a reduction in post-exercise blood flow in Duchenne patients [67]. Besides the central pathophysiological role of myofiber fragility, intrinsic satellite cell dysfunction also contributes to progressive muscle wasting via an impaired regenerative capacity of dystrophic muscles [45, 319].

In addition to a high frequency of micro-rupturing of the dystrophin-deficient sarcolemma and associated increases in cytosolic calcium levels and enhanced proteolytic degradation [144], the neuromuscular junction region was also shown to be more susceptible to contraction-induced injury, which results in impaired neuromuscular transmission [195, 244, 319]. Since the low-frequency versus high-frequency electro-stimulation pattern at the motor endplate dictates the slow-twitching versus fast-twitching properties within individual motor units [262, 263], a difference in the susceptibility of slow versus fast synaptic systems might play a key pathophysiological role in dystrophinopathy. Various neuromuscular disorders display a certain degree of fibre type specificity in muscle wasting [48, 81]. In Duchenne patients, subsets of fast myofibers appear to be more susceptible to initial degenerative processes as compared to a later onset of fibre wasting in slower-twitching fibre populations [367]. This correlates with an early decrease of fast myosin heavy-chain isoforms in dystrophic fibres [203, 259]. Indirect fast-to-slow transition processes were also observed in the DMD pig model of X-linked muscular dystrophy [104] and murine *mdx* skeletal muscle, whereby muscle transformation was shown to be linked to elevated levels of fibre respiration and enhanced protection from muscle damage [122]. Importantly, differential susceptibilities occur at the level of whole skeletal muscles. For example, extraocular muscles exhibit a very mild phenotype due to dystrophin deficiency [9].

### Sparing of naturally protected extraocular muscles in dystrophinopathy

Although dystrophin deficiency is clearly associated with highly progressive skeletal muscle degeneration, different subtypes of skeletal muscles are affected in distinctive ways. In contrast to severely degenerative limb and trunk muscles, extraocular muscles are spared from severe dystrophic changes [80, 100]. Unusual cell biological features of extraocular muscles include anatomical, biomechanical and functional compartmentalisation, myogenic processes driven by specific upstream activators, a longitudinal distribution of multi-terminal motor endplates along contractile fibres and morphologically distinct muscle spindles [63, 355]. The large variety of contractile patterns, including extraordinary fatigue resistance of fast fibres, within the extraocular muscle system is probably based on a broad distribution and expression pattern of key sarcomeric proteins, such as a large variety of slow and fast isoforms of light and heavy myosin chains [135]. A recent proteomic survey of the extraocular muscle proteome has established MyHC14 and MyHC15 as new markers of this subtype of skeletal muscles [106], in addition to the already-well-established super-fast myosin isoform MyHC13 [135].

The naturally protected phenotype of dystrophin-deficient extraocular muscles has been linked to a highly efficient calcium extrusion system, a specialised stem cell niche that provides efficient cellular regeneration and an enhanced remodelling capacity, the non-junctional upregulation of the dystrophin homologue utrophin Up-395 and concomitant rescue of dystrophin-associated glycoproteins, an enhanced cellular stress response, metabolic adaptations, the lack of fibrotic scarring and the relatively low load bearing of extraocular fibres [74, 106, 355, 378]. The fact that the contractile system surrounding the eyeball stays functionally unaffected in Duchenne muscular dystrophy is of considerable biomedical importance [9]. A better comprehension of the molecular and cellular processes that underlie the sparing of extraocular muscles during the course of disease progression in dystrophinopathy could be helpful for the identification of new therapeutic targets to counteract dystrophin deficiency, i.e. manipulation of calcium handling, utrophin replacement therapy, the targeted upregulation of molecular chaperones or improving the capacity for cellular regeneration.

### Reactive myofibrosis and chronic inflammation as key symptoms of dystrophinopathy

The disproportionate accumulation of proteins belonging to the extracellular matrix can be considered a key defining feature of dystrophinopathy [158, 254] and is probably closely linked to abnormal cellular signalling, extensive recruitment of the muscle repair machinery and chronic inflammation in the affected contractile tissues [309]. Chronic cycles of muscle tissue damage and fibre repair are triggered by dystrophin deficiency and cause a sustained immune response, which results in a chronic inflammatory phenotype of dystrophinopathy [280, 314, 315]. The innate immune response is accompanied by high levels of macrophage activity and the release of a variety of signalling factors, as well as the recruitment of myofibroblasts [31]. The potential interplay between chronic inflammation and myofibrosis in damaged fibres, as well as the role of abnormal Ca^2+^-handling in dystrophin-deficient fibres, is summarised in Fig. 6. Importantly, myofibroblasts exhibit an elevated synthetic capacity for the production of extracellular matrix proteins and therefore play a key role in reactive fibrotic changes in dystrophic muscle tissue.

The multi-functional cytokine named transforming growth factor TGF-β is released in large amounts from M2 macrophages and is involved in the activation of fibro-adipogenic progenitors [110] and other precursor cells in the muscle environment [20]. Enhanced activity of fibro-adipogenic progenitors plays a key role in dystrophinopathy. These precursors are resident in skeletal muscles and belong to the class of stromal cells that exhibit the potential to adapt to multiple cellular lineages [199]. Upon activation by fibre damage, fibro-adipogenic progenitors are involved in the generation of myofibroblasts, fibroblasts and adipocytes, as reviewed by Theret et al. [338]. Enhanced secretion of extracellular matrix components can then cause the excessive formation of fibro-fatty scars that surround contractile fibres and thereby negatively affect mechano-transduction and skeletal muscle elasticity [163]. In addition, adipogenic precursors were shown to be involved in interstitial remodelling, which is associated with disturbed adipogenesis [36]. This might explain the concomitant occurrence of reactive fibrosis and fat replacement in Duchenne muscular dystrophy causing major cellular complications for maintaining proper metabolic and contractile functions.

In one of the original descriptions of X-linked muscular dystrophy dating back to the year 1868, the French neurologist Guillaume-Benjamin-Amand Duchenne de Boulogne described an abundant production of fibrous tissue at the advanced stages of the disease and proposed to name this muscular disorder ‘paralysie myosclérosique’ (archived in [85]: Duchenne GB. Recherches sur la paralysie musculaire pseudo-hypertrophique ou paralysie myosclérosique. Archives générale médecine). The original medical description is certainly in agreement with the interstitial fibrotic phenotype of Duchenne muscular dystrophy. In normal skeletal muscles, the basal lamina and the extended layers of the extracellular matrix (consisting of the endomysium, perimysium and epimysium [379]) provide a supporting and signalling environment that forms protective sheets around contractile fibres [109]. Importantly, motor neurons and capillaries are embedded in the interstitial extracellular matrix for efficient neurotransmission within motor units and the steady supply of essential nutrients and oxygen to support the high bioenergetic needs of skeletal muscle metabolism.

Key proteins involved in structural maintenance of the extracellular matrix-sarcolemma axis via cell–matrix adhesion processes and the provision of mechanical support and lateral force transmission include various (i) collagen isoforms (Col I, III, IV, V and VI), (ii) a large number of proteoglycans (biglycan, prolargin, mimecan, decorin, asporin, fibromodulin, perlecan, syndecan, lumican, agrin and aggrecan), (iii) matrix crosslinkers (fibronectin), (iv) non-architectural matricellular proteins (periostin, osteopontin, dermatopontin and nidogen), (v) matrix metalloproteinases (MMP 1, 2, 9, 10 and 13) and their inhibitors named tissue inhibitors of metalloproteinases (TIMP) and plasminogen activators (PA), (vi) integrin (α7β1-integrin), (vii) laminin (α2β1γ1 laminin-211) of the basal lamina, (vii) signalling proteins (myokines and growth factors) and (vii) the dystroglycan complex with its laminin-binding component alpha-dystroglycan [109, 141, 254, 379]. Disturbances of adhesion receptors and rearrangements of structural fibres within the extracellular matrix play an important pathophysiological role in many neuromuscular pathologies, including X-linked muscular dystrophy [158]. Since the extracellular matrix is intrinsically involved in the maturation and differentiation of muscle fibres and adaptive fibre transitions, myofibrosis has a considerable influence on the loss of regenerative capacity in dystrophic muscles [163]. The loss of tissue elasticity and progressive cellular scarring causes decreased mechano-transduction and impaired skeletal muscle function, and may also affect crosstalk between muscle and tendon [375].

As indicated in Fig. 6, a large number of extracellular matrix proteins were shown to be drastically increased or modified in their isoform expression pattern in both patients and animal models of X-linked muscular dystrophy, including collagens, proteoglycans, adhesion receptors, matricellular proteins and matrix metalloproteinases [7, 92, 309, 344]. Systematic large-scale surveys using mass spectrometry–based proteomics identified a drastic increase in the matricellular proteins dermatopontin [41] and periostin [138], as well as the extracellular matrix stabilisers biglycan and fibronectin [230, 239] in conjunction with elevated collagen expression in the highly fibrotic- and dystrophin-deficient diaphragm muscle [141]. Especially striking are high levels of the cross-linking enzyme lysyl oxidase and concomitant increases in the level of cross-linked collagen clusters in the disturbed extracellular matrix of the dystrophic diaphragm [310]. These findings were confirmed by a longitudinal study of histological changes during progressive muscle wasting in Duchenne patients, which clearly established that endomysial fibrosis presents the most significant myopathological feature in correlation to the gradual loss of motor functions [64]. It is therefore not surprising that collagens detected by advanced imaging technology have been suggested as novel biomarkers for dystrophinopathy [272]. Primary fibroblasts isolated from Duchenne patients produce elevated levels of decorin and collagen, and are characterised by elevated proliferation rates [376, 377]. This pro-fibrotic phenotype shows high sensitivity to transforming growth factors, which agrees with the finding that muscular dystrophy-associated fibrosis is driven by the transforming growth factor TGF-β-related activation pathway [196, 208, 356]. Interestingly, the serum- and glucocorticoid-inducible kinase SGK1 appears to be a fibrosis-stimulating factor that plays a role in fibrotic remodelling and muscular weakness [321].

These findings of dysregulated matricellular proteins and elevated collagen fibrillogenesis due to hyperactive fibroblast populations are crucial for our general understanding of the detrimental role of myofibrosis in X-linked muscular dystrophy. Skeletal muscles appear to be capable to swiftly address minor acute injuries by upregulating repair mechanisms and the regenerative activation of myoblasts and protective involvement of fibroblasts [379]. However, chronic muscle wasting seems to overwhelm the beneficial effects of elevated activity levels of the connective tissue and causes instead fibrotic tissue scarring [141, 254]. As discussed in the below section on novel treatment options for dystrophinopathies, it will be crucial to counteract the progressive nature of fibrotic changes in order to increase the chance for the successful application of new cell-mediated or gene-based therapies [295]. However, a recent study on dystrophinopathy-associated fibrosis has shown different degrees of altered muscle stiffness and collagen amounts in *extensor digitorum longus* versus *soleus* muscles [34], suggesting the selective targeting of the alignment of large collagen structures rather than total collagen for the most efficient treatment of fibrotic stiffness in X-linked muscular dystrophy [309, 310].

The inflammatory phenotype of progressive skeletal muscle degeneration due to dystrophin deficiency is associated with a variety of cellular immune responses [280], especially interference by the innate immune system [31] but also acquired immune responses [314]. As already discussed above, there appears to be a close link between chronic inflammation and reactive myofibrosis in X-linked muscular dystrophy [20]. Resident immune cells play an important role in normal skeletal muscle homeostasis. Of note, a resident macrophage population associated with the epimysium and perimysium space between contractile fibres can swiftly act and remove cellular debris. In muscular dystrophy, the conversion of activated monocytes and the invasion by large numbers of additional macrophages is a key feature of muscle membrane lesions [358, 359]. Besides M1 and M2 macrophages, the promotion of the degenerative phenotype seen in dystrophic muscle fibres includes additional immune cells, including helper CD4 + T lymphocytes, cytotoxic CD8 + T lymphocytes, eosinophiles and neutrophiles as first responders of inflammation, as well as infiltration of the dystrophic muscle by myeloid cell populations [314, 315].

High levels of contraction-induced injury due to dystrophin deficiency cause fibre disintegration, and the cellular damage to the weakened sarcolemma is associated with damage-associated molecular patterns (DAMPs) and the release of typical DAMP components such as nucleic acids and ATP molecules [219]. DAMP molecules are recognised by the innate immune system and result in an inflammatory response with a central role played by the nuclear factor NF-κB and the inflammasome [280]. In addition, the shedding of peptides, protein fragments or proteins through the leaky muscle plasmalemma presents these muscle components as potential neoantigens to the adaptive immune system [341]. Importantly, a large number of signalling cytokines and chemokines promote the infiltration of dystrophic fibres by neutrophiles, macrophages and dendritic cell populations [31, 319].

The recent proteomic analysis of the role of the spleen in X-linked muscular dystrophy confirmed pathophysiological crosstalk between dystrophic muscles and the secondary lymphoid organ system [76]. The spleen, which is majorly involved in antigen detection, antibody production and the removal of abnormal erythrocytes, exhibits morphological adaptations of lymph nodes in its white pulp region due to dystrophin deficiency [293]. Importantly, muscular dystrophy is associated with changed numbers of splenic inflammatory monocytes and an increased migration pattern of immune cells from the splenic reservoir to damaged contractile fibres [219, 220, 256]. The movement of splenic monocytes and differentiation into macrophages seems to be crucial for promoting chronic inflammation in dystrophin-deficient skeletal muscles [277]. These major changes due to inflammatory mechanisms in dystrophic fibres are summarised in Fig. 6.

### Late-onset cardiorespiratory pathophysiology in dystrophinopathy

Impaired cardiorespiratory function plays a key pathophysiological role in X-linked muscular dystrophy, especially in the second decade of life of Duchenne patients [25], and requires mechanical ventilatory support to manage respiratory insufficiency [32] and cardiac drug treatment including angiotensin-converting enzyme inhibitor therapy [161, 216]. Right and left diaphragmatic motions are reduced following inspiration [93], and progressive respiratory decline is already present during the early ambulatory phase in Duchenne patients [276]. Imaging studies of respiratory muscle movements revealed a drastically reduced thoracic cavity area and reduced chest wall contraction during inspiration-expiration patterns in muscular dystrophy, as well as increased fat infiltration in accessory respiratory muscles [17]. The progression of myocardial fibrosis in Duchenne and Becker muscular dystrophy patients is associated with poor prognosis. Thus, in analogy to skeletal muscle fibrosis and its detrimental effect on motor function [163, 272], excess accumulation of extracellular matrix components also plays a crucial role in progressive cardiomyopathic complications in dystrophinopathy.

**Fig. 7:**
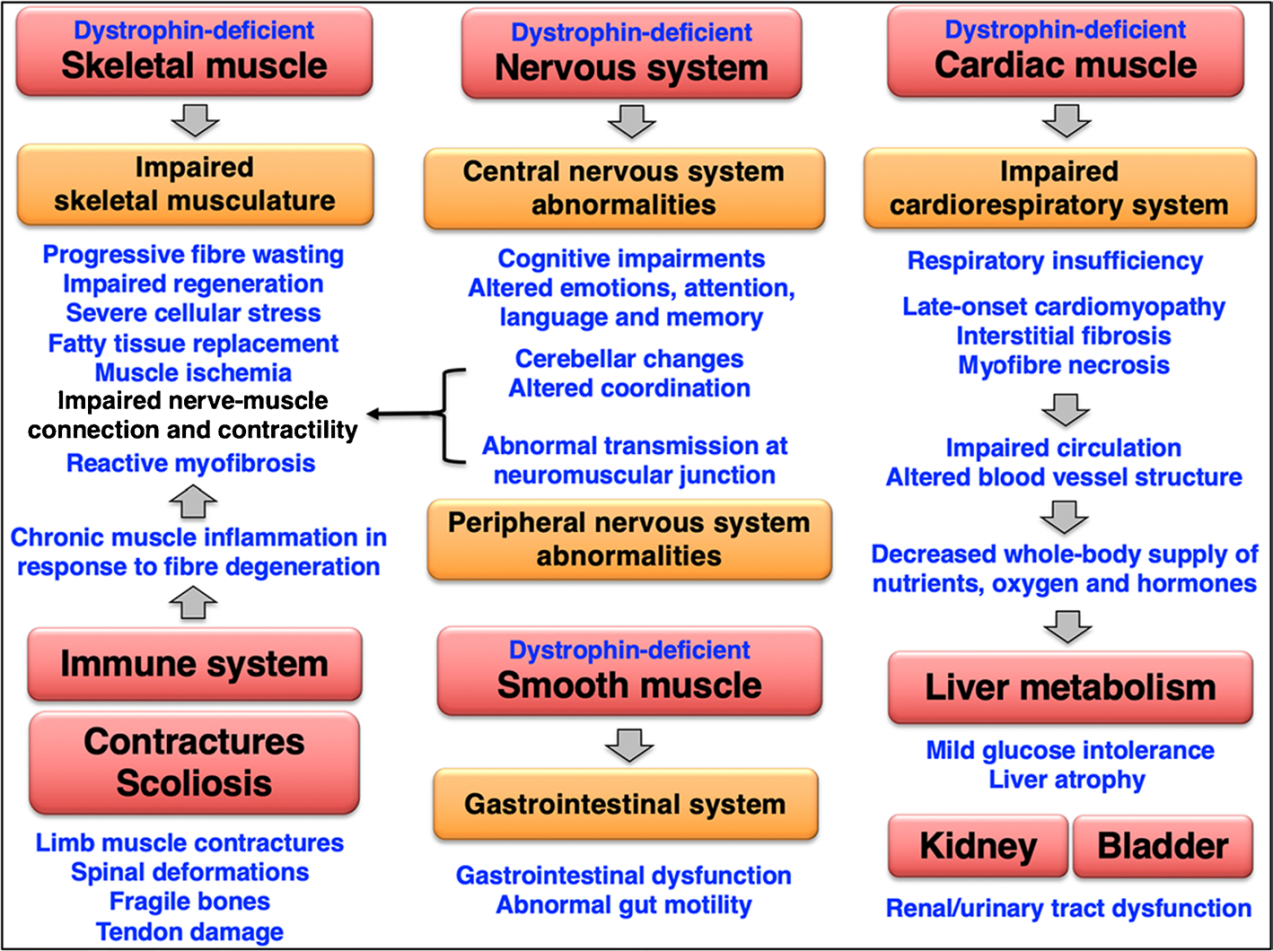
Overview of skeletal muscular degeneration, multi-systems pathophysiology and organ crosstalk in Duchenne muscular dystrophy

Cardiomyocytes, which only exhibit limited regenerative capacity, do not undergo extensive degeneration-regeneration cycles in muscular dystrophy. The central importance of the cardio-respiratory syndrome and interconnectivity of body-wide effects of dystrophin deficiency is diagrammatically summarised in Fig. 7, which gives an overview of the complexity of abnormalities in the skeletal musculature, the central and peripheral nervous system, smooth muscles and the cardiovascular system, as well as potential organ crosstalk that is related to secondary dysfunctions in the liver and the renal-urinary tract. Interstitial fibrosis and myofiber necrosis cause cardiac weakness which in turn negatively affects efficient circulation. Thus, dystrophinopathy-associated cardiomyopathy is probably indirectly linked to a decreased whole-body supply of nutrients, oxygen and hormones in X-linked muscular dystrophy. Chronic impairment of circulation might be involved in fatty liver disease, as discussed in more detail in the below section on organ crosstalk. Both the long-term impairment of the circulatory system and chronic exposure to released intracellular molecules from damaged skeletal muscle fibres may also contribute to renal abnormalities in dystrophinopathy [172].

In analogy to skeletal muscle, full-length dystrophin also forms a tight complex with glycoproteins in the heart, where it occupies a major mechanical, protective and signalling role in the organisation and maintenance of the surface membrane system of cardiomyocytes [154, 162]. In conjunction with the talin-integrin system, the dystrophin complex associates with vinculin at costamers in a perpendicular direction to the longitudinal axis of cardiac muscle cells [56]. However, in contrast to skeletal muscle, the core cardiac dystrophin-glycoprotein complex exhibits a differential composition in relation to dystrobrevins and syntrophins and does not display a direct link to the nNOS isoform of nitric oxide synthase. The cardiac dystrophin complex features additional linkages to the molecular chaperone alphaB-crystallin, the large scaffolding phosphoprotein ahnak-1, the caveolae component cavin-1 and the cardiac cytoskeletal component cypher [152].

Important aspects of the molecular and cellular pathogenesis of dystrophinopathy-associated cardiomyopathy were determined by studying dystrophic mouse and pig models [139, 232, 331]. Systematic proteomic surveys of the dystrophin-deficient heart have revealed drastic changes in the dystrophin-associated glycoprotein complex [114], which in turn triggers an abnormal expression pattern of proteins involved in cytoskeletal networks, the extracellular matrix, the cardiac contractile apparatus, energy metabolism, signalling mechanisms and the cellular stress response [142]. On the subcellular and molecular level, the dystrophic heart is primarily characterised by sarcolemmal disintegration and significantly reduced levels of laminin, nidogen and annexin [232]. Pathophysiological patterns of cardiac fibre necrosis, interstitial inflammation and reactive myofibrosis result in heart disease in the majority of patients afflicted with Duchenne muscular dystrophy [216].

The fibrotic phenotype of the dystrophin-deficient heart resembles some of the alterations that occur during skeletal muscle fibrosis, including increased activity of pro-fibrotic genes, collagen accumulation, enhanced activity of connective tissue growth factors and heightened transforming growth factor TGF-β signalling via coronary endothelial cells [142, 148]. Workload-induced cardiomyocyte injury and the acute elevation of mechanical stress in the dystrophic heart is also linked to abnormal Ca^2+^ handling [175]. Fragility of the dystrophin-deficient plasma membrane and altered ion homeostasis is clearly associated with a lowered luminal Ca^2+^-buffering capacity of cardiomyocytes, hypersensitive excitation–contraction coupling and the activation of the stretch-activated Ca^2+^-influx pathway via transient receptor potential vanilloid channels of type TRPV2 [189, 192, 302].

### Neurological complications in dystrophinopathy

The multi-system pathophysiology of Duchenne muscular dystrophy is characterised by a variety of neurological complications [210]. However, mental issues do not appear to be progressive and were shown not to correlate with the severe loss of motor function in dystrophic patients. Different degrees of neurodevelopmental, psychiatric, behavioural and emotional symptoms have been clearly established in a subset of individuals suffering from severe forms of dystrophinopathy [61] but can also occur in more benign cases of Becker muscular dystrophy [173]. Neurological issues are characterised by social, behavioural and emotional problems [129, 275], delayed milestones of language development [58], adaptive deficiencies [57], impaired working memory [336] and a variety of neuropsychiatric diseases such as hyperactivity, obsessive–compulsive behaviour, attention deficit and autism spectrum disorders [128, 257, 258]. However, it is important to emphasise that severe cognitive impairments and mental retardation occur only in a subgroup of patients and were shown to be secondary in nature to physical handicap [210, 311].

Non-progressive cognitive deficiencies in Duchenne patients were shown to correlate at least partially with the type of mutation in the *DMD* gene and accompanying effects on the expression levels of the various dystrophin isoforms in the central nervous system [69, 337]. Especially abnormalities in the dystrophin isoform Dp140 [16, 95] appear to be associated with severe forms of cognitive defects [18, 347]. Neuronal dystrophins include the short forms Dp45 and Dp71, the medium-sized isoform Dp140 and full-length Dp427 [228, 240]. Brain-associated proteoforms of Dp427 were shown to feature biochemical properties that are typical of cytoskeletal components and are similar to the actin-binding protein Dp427 present in skeletal muscles [96, 97]. The large Dp427 isoforms localise to neurons of the hippocampus and the cerebral cortex [159, 181, 348]. During the development of the central nervous system, the Dp140 isoform is most highly expressed in the brain [183]. However, following brain maturation, the most abundant dystrophin is represented by isoform Dp71 [240] that is mostly localised in neuronal and glia cells of the olfactory bulb and the dentate gyrus [54, 329]. In analogy to skeletal muscles, various dystrophin isoforms in the brain are linked to glycoproteins such as dystroglycans and sarcoglycans [55, 364]. Dystrophin-glycoprotein complexes were shown to be associated with key neuronal processes, including modulation of synaptic activity, excitability, plasticity and integration of signalling cascades [125, 260].

Abnormalities in brain white matter were recently established in children afflicted with dystrophinopathy by diffusion tensor imaging, a magnetic resonance imaging technique that is suitable to especially determine the axonal organisation within the brain [265]. These neurological complications seem to be connected to altered developmental pathways in the cerebellum resulting in disturbed cerebro-cerebellar loops [59]. Besides studying brains from Duchenne patients by mostly non-invasive technologies, crucial insights into complex neurological changes have been generated by studying molecular and cellular brain abnormalities in animal models of dystrophinopathy. One of the most widely employed dystrophinopathy models, the *mdx* mouse, exhibits deficits in long-term consolidation memory, characteristic changes in associative learning patterns and bioenergetic alterations in distinct brain regions [227, 343, 351] making it a suitable system for detailed neurochemical and cellular studies. Important findings include the establishment of disturbed interactions between glial and endothelial cells at the blood–brain barrier [246], an abnormal neuronal receptor density in Purkinje cells [169] and the central role of impaired cerebellar function as highlighted by disrupted circuit signals between Purkinje cells and the cerebellar nuclei in the dystrophin-deficient brain [320].

The altered blood–brain barrier function in the *mdx* brain is associated with a reduction in dystrophin-associated glycoproteins [247] and upregulation of the matrix-metalloproteinases MMP2 and MMP9 [245]. Interestingly, the proteomic analysis of the *mdx-4cv* mouse model revealed increases in vimentin and annexin that might be associated with the cytoskeletal stabilisation of dystrophin-lacking brain cells and enhanced membrane repair processes [235]. Elevated expression levels of the glial fibrillary acidic protein, which is an established marker of astrogliosis due to its unique localisation to astrocytes in the central nervous system [136], were also demonstrated to occur in the *mdx-4cv* brain by mass spectrometry, immunoblotting and immunofluorescence microscopy [235]. This agrees with the reported occurrence of multifocal glial nodules in the brain of a Duchenne patient with severe mental retardation [150]. Increases of glial fibrillary acidic protein indicate ongoing neurodegeneration-associated astrogliosis with accumulation of this intermediate filament component in the brain lacking certain dystrophin isoforms, such as Dp427 and Dp140.

### Impaired energy metabolism, abnormal liver function and gastrointestinal abnormalities in dystrophinopathy

The recent comparative proteomic profiling of muscle biopsy samples from Duchenne patients, as compared to healthy controls, has demonstrated metabolic disturbances both at the level of anaerobic pathways and lipogenesis [38]. Since skeletal muscle tissue plays a crucial role in the interorgan crosstalk of metabolic regulation [10] and because of a close relationship between nutritional uptake by the gastrointestinal tract, liver metabolism and skeletal muscle function, dystrophinopathy-associated changes that affect the efficient crosstalk between these vital organs are collectively discussed in this section. The combined skeletal musculature is a key organ system involved in the integration of carbohydrate, lipid and protein metabolism [10]. The absorption of digested nutrients through the gastrointestinal tract leads to the transportation of vital biomolecular building blocks via the circulatory system towards muscle, adipose and liver tissue. Contractile fibre protein forms an essential primary amino acid reservoir for the regulation of whole-body protein. This is crucial for substrate provision to maintain protein synthesis during periods of starvation and disease [286]. In addition, muscle tissues serve as the most abundant primary location for insulin-dependent glucose uptake throughout the body and storage of glycogen for utilisation within skeletal muscles. The shuttling of lactate and glucose between muscle and liver, also known as the Cori cycle, is a key interorgan pathway that links anaerobic skeletal muscle glycolysis with liver metabolism [313]. The interrelationship between liver, adipose tissue and skeletal muscles also determines the rate of fatty acid transportation and the regulation of oxidative metabolism [87].

A variety of muscular dystrophies are associated with a considerably perturbed skeletal muscle metabolism in both the early and acute phase of these disorder [318], including changed lipid utilisation and impaired energy metabolism in Duchenne patients [60, 176, 317]. Pathological developments in Duchenne children are not only characterised by highly progressive muscle wasting, but also delayed growth resulting in relatively short stature and increased fat mass. The altered muscle-to-fat ratio and overall body mass alterations in dystrophinopathy cause a high prevalence of obesity in Duchenne children and these body mass changes are linked to metabolic disturbances [294]. This in turn has an impact on bioenergetic requirements and nutritional status. While weight gain due to corticosteroid therapy might require weight management in younger Duchenne patients, issues with malnutrition [94] could necessitate supplemental feeding regimes at more advanced stages of the disease [26, 62]. The implementation of nutritional interventions, including micronutrient supplements such as calcium, creatine and vitamins, is a potential way to address certain aspects of bioenergetic dysregulation and loss of motor function due to progressive fibre degeneration [288].

Disturbed metabolism in X-linked muscular dystrophy is associated with abnormal calcium handling and mitochondrial dysfunction, reduced ATP levels, enhanced phosphorylation of the AMP-activated protein kinase and accumulation of reactive oxygen species [126]. Abnormal bioenergetic processes are also observed in dystrophic animal models including defective regulation of metabolic pathways [91]. Substrate-selective limitation of biochemical reaction rates of anaerobic metabolism in fast muscles and aerobic metabolism in slow muscles were shown to be associated with decreased oxidative utilisation of both glucose and free fatty acids [184, 322]. Excellent indicators of disturbed fatty acid metabolism are the various isoforms of fatty acid binding protein (FABP) [78]. A large number of proteomic surveys have established a reduced abundance of the FABP3 (H-FABP) isoform in various dystrophic skeletal muscles and the heart [116, 138, 230, 232, 239] and concomitant increase in serum [123, 231, 316]. In contrast to contractile tissues, the liver exhibits increased levels of the FABP5 (E-FABP) isoform of fatty acid protein in X-linked muscular dystrophy [236]. Elevated amounts of liver-associated FABP5 suggests changes in the binding of long-chain fatty acids in dystrophinopathy and agrees with fatty liver disease and ectopic fat accumulation being associated with the dystrophic phenotype [78]. This agrees with elevated serum levels of the liver damage marker alanine aminotransferase in the majority of Duchenne patients [167].

Besides skeletal and cardiac muscle, the dystrophin isoform Dp427 and its associated glycoprotein complex are also present in smooth muscle cells [120, 134]. A recent proteomic survey of the stomach wall identified dystrophin Dp427-M in association with alpha/beta-dystroglycan, alpha/beta/delta-sarcoglycan, gamma/epsilon-sarcoglycan, alpha/beta-dystrobrevin and alpha1/beta1/beta2-syntrophin [77]. The interface between the pancreas and the stomach of the *mdx-4cv* mouse model of X-linked muscular dystrophy wall was confirmed to be characterised by a loss in dystrophin and reduced abundance of sarcoglycan and dystroglycan, in addition to lower expression levels of the extracellular matrix component laminin, the sarcomeric protein titin and the actin-binding protein filamin [77]. The DMD pig model of muscular dystrophy also exhibits abnormal digestion and absorption capacity in the gastrointestinal tract [384]. The associated patterns of malnutrition are in agreement with gastrointestinal dysfunction in the dystrophic phenotype [26, 177, 186, 200].

### Abnormal kidney and bladder function in dystrophinopathy

The kidney contains the Dp140-B/K dystrophin isoform and the shorter Dp71 proteoform of dystrophin [182] and dystrophin-associated proteins are present at relatively high abundance in renal cells [83, 119]. Dystroglycans were shown to play a crucial role during kidney epithelial morphogenesis [86]. However, in contrast to contractile fibres, the dystrophin-related protein β-dystrobrevin appears to occupy the central position in the anchoring of dystrophin-associated proteins in non-muscle tissues instead of dystrophin [187]. These dystrobrevin complexes were found to be present in endothelial cells and the basal region of renal epithelial cells [188]. The smooth muscle system and afferent nerve fibres of the bladder express the short dystrophin isoforms Dp71 and Dp140 [185]. Dystrophin expression might therefore be primarily affected in the bladder and kidney of patients with certain mutations in the *DMD* gene [28, 99, 243], or these organ systems are altered due to secondary and body-wide adaptations.

With advancing age, the prevalence of urological manifestations increases in Duchenne patients [11, 172]. However, relatively little is known about the pathophysiological mechanisms of bladder smooth muscle dysfunction and urinary incontinence and their full clinical extend in patients afflicted with dystrophinopathy [200, 222]. In contrast, a variety of studies have characterised renal dysfunction in X-linked muscular dystrophy. Duchenne patients at advanced stages of muscular dystrophy are especially susceptible to kidney disease [223], including fatal cases of acute renal failure [207]. Kidney disease was shown to also correlate with cardiomyopathic complications causing in some cases the cardio-renal syndrome [357]. Symptomatic nephrolithiasis [303, 308], abnormal filtration rates [33] and impaired kidney perfusion [223] were shown to occur in large cohorts of non-ambulatory Duchenne patients.

It is possible to monitor the dysfunction of the kidney by routine clinical tests, such as the cystatin C–estimated glomerular filtration rate [357, 361]. Studies of the dystrophic *mdx*-type mouse models revealed cellular changes in the kidney [118] and reduced renal function [363] and were also instrumental to test for toxic side effects on the kidney due to drug treatment or experimental exon-skipping therapy [270, 381]. The systematic identification of proteome-wide changes in the kidney has demonstrated the increased expression levels of the FABP1 (L-FABP) isoform of fatty acid–binding protein [83]. Changes in this crucial metabolic protein [145] are most likely associated with ectopic fat deposition and chronic renal dysfunction [78, 373].

Disease processes occurring in the kidney and bladder can be studied completely non-invasively by analysing changes in biomarkers in urine specimens [153, 370]. Urine displays a highly complex metabolome and proteome with a large number of biomolecules that exhibit excellent diagnostic and prognostic properties [191, 369, 382]. However, it is difficult to establish bladder- or kidney-specific tissue damage markers [380]. The most drastic proteomic or metabolomic changes in urine samples from Duchenne patients are linked to muscle-specific or body-wide alterations, i.e. the presence of muscle titin fragments [107, 205, 283], high levels of ferritin [282] and an elevated concentration of the prostaglandin tetranor-PGDM [330]. An exception is the kidney-specific protein named uromodulin, which is exclusively produced in the ascending limb of the loop of Henle and in the distal tubular region of the nephron. Uromodulin is highly abundant in urine and a marker of chronic renal disease [143, 217] and was identified to be significantly increased in urine from Duchenne patients [283]. This indicates considerable renal injury in dystrophionapthy. Another striking feature is enhanced oxidative damage to urinary proteins, which is proposed to be generated by oxidant hypochlorous acid via the enzyme myeloperoxidase from neutrophils [333].

### Novel proteomic biomarkers of the complex aetiology of Duchenne muscular dystrophy

X-linked muscular dystrophy is characterised by a highly complex process of skeletal muscle wasting and secondary pathophysiological effects that are reflected by considerable levels of multi-systemic change and organ crosstalk [84, 111, 213]. This body-wide aetiology requires multi-disciplinary approaches for the optimum diagnosis, prognosis and management of dystrophinopathy [25–27, 340]. Although standard serum enzyme assays used for the routine evaluation of skeletal muscle damage, such as creatine kinase or myoglobin tests, are useful indicators of tissue changes and the release of intracellular muscle content, these analytes are not specific enough for the monitoring of neuromuscular disease progression and therapeutic impact [250]. Thus, there is an urgent clinical need for the establishment of novel biomarker signatures that are suitable for the proper differentiation between muscle-related degeneration versus non-muscle-associated abnormalities in Duchenne patients, as well as monitoring of signalling events between different tissues and organ systems [79]. Improved diagnostic and prognostic tools would help to better predict the complexity of disease progression in relation to fibre necrosis, reactive myofibrosis, fat substitution and sterile inflammation. Therapeutic biomarkers should be able to evaluate both intended outcomes versus adverse effects of innovative therapeutic approaches at the genetic, pharmacological and cellular level [82, 237, 250]. As reviewed by Szigyarto and Spitali [328], biomarkers in the field of dystrophinopathy can be divided in relation to susceptibility, screening, diagnosis, prognosis, prediction, therapy monitoring and safety.

Clinical assays based on marker molecules, such as DNA, miRNA, metabolites, lipids and/or proteins [43, 146, 168], should on the one hand be highly specific, robust, sensitive and cost-effective and on the other hand exhibit ideally only a minimum susceptibility to interference by gender, age, ethnicity, nutrition, lifestyle and circadian rhythm [250]. User-friendly biomarker tests could be highly useful to improve prenatal analysis, new-born screening and estimating disease initiation in order to decisively reduce the time between observation of initial symptoms and consolidated differential diagnosis [156, 281]. The establishment of sets of prognostic and predictive biomarkers would allow the monitoring of pathological progression, potential adverse clinical events, the differential screening of patient populations and the determination of the sensitivity of individual patients to new treatment protocols [328]. While body-wide responses to new drug treatments can be examined by pharmacodynamic markers and potential cytotoxic effects by safety markers, the repeated assessment of improved disease status and possibly convalescence should be enabled by therapy-monitoring marker molecules [1, 237].

Besides systematic transcriptomic surveys [190, 306] and metabolomic research [60, 178, 345] in the field of dystrophinopathy, a major area of omics-based biomarker discovery employs mass spectrometric and proteomic screening protocols for the large-scale identification of new peptides, protein fragments and protein biomarker candidates [42, 82]. A few studies have also used multi-omics approaches for studying dystrophic changes at the various levels of biological organisation from gene to mRNA to protein expression [122, 224, 352]. The integration of genomics and proteomics for the in-depth proteogenomic characterisation of rare neuromuscular disorders is an attractive approach that combines advanced proteomic screening with gene discovery [279].

The initial experimental design of mass spectrometry-based proteomic workflows should ideally take into account the following: (i) an optimised experimental and bioanalytical design for tissue preparation, sample collection, specimen storage, subcellular fractionation and protein extraction prior to biochemical analysis; (ii) a suitable proteomic discovery strategy (i.e. a specific mass spectrometric technique and labelling method) following large-scale protein separation using either bottom-up (liquid chromatography) or top-down (two-dimensional gel electrophoresis) approaches; (iii) the unequivocal demonstration of disease/therapy-associated differential protein expression patterns using advanced bioinformatic analysis of proteomic data; (iv) the independent verification of the specificity, sensitivity and robustness of new protein biomarker candidates using standardised biochemical, immunochemical, molecular biological, cell biological and physiological assays; and finally (v) a preliminary prioritisation of the most promising biomarker signature for the tissue/biofluid-specific evaluation related to specific goals such as improved screening, diagnosis, prognosis, pharmacodynamics or therapy monitoring [82]. Following the identification of new peptides or proteins using patient biopsy or biofluid material, cell culture or animal models, new biomarker candidates undergo then an intense validation process using retrospective or prospective investigations to test their validity and significance prior to clinical usage [1].

The skeletal muscle proteome [37] consists of at least 10,000 proteoforms [65, 66, 229], and systematic screening of changes in both muscle-associated proteins and released proteoforms in biofluids has identified a large number of potential biomarker candidates [42, 82, 124, 237]. This includes significant expression changes in skeletal muscle proteins that are involved in ion homoeostasis, cellular signalling cascades, the regulation of excitation–contraction coupling, the sarcomeric units and the maintenance of contraction-relaxation cycles, the formation of the extracellular matrix, the stabilisation of the cytoskeletal network, the cellular stress response, metabolism and bioenergetics in X-linked muscular dystrophy [38, 137, 138, 140, 230, 239, 269, 353]. Abnormal protein expression was also documented in the heart, stomach, brain, liver, kidney and spleen of the dystrophic phenotype [77, 83, 232, 235, 236]. However, muscle tissue–associated markers are only of limited usefulness for routine and repeated sampling approaches. In order to avoid unnecessary complications due to highly invasive tissue biopsy procedures, instead suitable and representative biological fluids can be tested for the presence of disease markers.

Proteomic serum profiling has been successfully applied to study inflammation, mitochondrial abnormalities, membrane instability and fibrosis in muscular dystrophy [123, 124, 231, 237]. While serum and plasma specimens are harvested by minimally invasive methods, biofluids such as saliva or urine have the advantage of being sampled in a completely non-invasive way. Both biological fluids contain large and complex proteomes that are suitable for the routine diagnostic analysis of body-wide health status [107, 238]. Promising new biofluid markers of Duchenne muscular dystrophy include fibronectin, titin fragments, fatty acid–binding protein FABP3, malate dehydrogenase MDH2, the inflammation-inducible plasma marker haptoglobin, carbonic anhydrase CA3, myosin light-chain MLC3 and matrix metalloproteinase MMP9 [14, 168, 194, 231, 305, 323]. Some of these markers can now be validated in a number of new therapeutic applications [5].

### Therapeutic implications and future perspectives

New treatment approaches to ameliorate the dystrophic phenotype include (i) pharmacological interventions using drugs that modulate the immune response and inflammation, abnormal ion homeostasis, impaired excitation–contraction coupling, cellular growth patterns, abnormal metabolic pathways, cholesterol metabolism, oxidative stress and cardio-respiratory complications [8, 132, 155, 209, 214]; (ii) myoblast transfer therapy [15, 225, 304]; (iii) stem cell therapy [24, 40, 325]; (iv) somatic genome editing using CRISPR/Cas9-mediated exon excision [12, 171, 218]; (v) heat shock protein induction to enhance the natural cellular stress response provided by molecular chaperones [108, 334]; (vi) stop codon read-through therapy [127, 264, 326]; (vii) vector transfer therapy [121, 242, 296]; (viii) exon-skipping therapy [49, 131, 180]; (ix) electrical nerve stimulation to induce muscle transitions [122]; and (x) utrophin substitution therapy [193, 312, 362]. An interesting approach is the repurposing of established pharmacological substances and testing of multi-drug combinations in experimental trials using genetic animal models of Duchenne muscular dystrophy [383].

Detailed discussions of the clinical advantages versus potential limitations of these new treatment options and their current validation status in preclinical or clinical studies have been published [101, 299, 354]. Recently, several novel compounds have emerged which have been approved or await final approval by medicines agencies. This includes the FDA-approved novel corticosteroid Deflazacort that was shown to be associated with improved muscle strength [267] and agents with conditional approval such as Casimersen for skipping exon 45 resulting in the elevated production of dystrophin in skeletal muscle [301] and Eteplirsen for skipping of exon 51 which causes delayed loss of ambulation in some patients [211] and Golodirsen and Viltolarsen for skipping exon 53 resulting in increased dystrophin levels [49, 102], as well as the oxadiazole drug named Ataluren, approved by the European Medicines Agency, which is supposed to help restore dystrophin by supressing nonsense mutations [22]. Ongoing clinical evaluations of further pharmacological interventions that focus on skeletal muscle abnormalities in dystrophinopathy include the anti-inflammatory substances Valmorolone and Cosyntropin, the anti-fibrotic drugs Givinostat and Pamrevlumab and the myostatin inhibitor RO7239361 [284, 298].

Since reactive myofibrosis plays such an important pathophysiological role in muscular dystrophy [64, 141, 254], the prevention, halting or reversal of fibrosis is a key factor for avoiding complications and providing optimised conditions prior to the application of novel therapeutic approaches, such as gene transfer or interventions with stem cells [163, 295]. A potential way to address the issue of excess tissue scarring would be to drastically decrease the expression levels of pro-fibrotic proteins via antisense oligonucleotide therapy [201].

## Conclusions

Although Duchenne muscular dystrophy is primarily defined as a skeletal muscle wasting disorder, its pathophysiological changes also affect multiple non-muscle tissues and organ systems. This makes dystrophinopathy a multi-systems disorder with complex disturbances of whole-body homeostasis. Hallmarks of both sequential and overlapping alterations due to deficiency in dystrophin include (i) progressive skeletal muscle degeneration in association with fat replacement, chronic inflammation and reactive fibrosis; (ii) scoliosis, joint deformation and contractures; (iii) respiratory insufficiency; (iv) late-onset cardiomyopathy; (v) neurological deficiencies that may cause cognitive impairments, emotional issues and attention deficit; (vi) endocrine, metabolic and bioenergetic disturbances; (vii) gastrointestinal dysfunction; (viii) fatty liver disease; and (ix) renal and urinary tract dysfunction. Therefore, for the future development of new treatment approaches, such as gene therapy, as well as the establishment of novel biomarkers to improve differential diagnosis, prognosis and therapy monitoring of Duchenne muscular dystrophy, both multi-system pathology and organ crosstalk should be taken into account. In order to decisively increase the long-term survival of Duchenne patients, new combinations of pharmacological therapy, cellular interventions and gene substitution approaches should be designed that can be employed together with physiotherapy and optimum nutritional support to address the complex and body-wide pathology of dystrophinopathy.
